# Ablation of hepatic malignant tumors with irreversible electroporation: A systematic review and meta-analysis of outcomes

**DOI:** 10.18632/oncotarget.14030

**Published:** 2016-12-20

**Authors:** Guo Tian, Qiyu Zhao, Fen Chen, Tian’an Jiang, Weilin Wang

**Affiliations:** ^1^ State Key Laboratory for Diagnosis and Treatment of Infectious Diseases, The First Affiliated Hospital, Zhejiang University School of Medicine, Hangzhou 310003, China; ^2^ Collaborative Innovation Center for Diagnosis and Treatment of Infectious Diseases, The First Affiliated Hospital, Zhejiang University School of Medicine, Hangzhou 310003, China; ^3^ Department of Ultrasound Medicine, The First Affiliated Hospital, Zhejiang University School of Medicine, Hangzhou 310003, China; ^4^ Department of Hepatobiliary and Pancreatic Surgery, The First Affiliated Hospital, Zhejiang University School of Medicine, Hangzhou 310003, China

**Keywords:** irreversible electroporation, tumor, alkaline phosphatase, aspartate aminotransferase, bilirubin

## Abstract

**Background:**

Irreversible electroporation (IRE) ablation is a new technique that is used to eliminate malignant tumors through nonthermal approaches.

**Objective:**

The purpose of this review was to evaluate the efficiency of IRE for hepatic malignant tumors.

**Methods:**

A systematic search was performed from PubMed, Embase, Web of science, Scopus and other potential literatures from references in relevant articles July 26th, 2016. Overall estimates of pooled standard mean difference (SMD) with 95% confidence interval (CI) were calculated for the changes of the pre- and post-IRE longest diameter, alkaline phosphatase (ALP), aspartate aminotransferase (AST) and serum total bilirubin levels. Sensitivity analysis and publication bias and were performed after the pooled analysis, and the quality of the included literatures was appraised using Newcastle-Ottawa Scale (NOS).

**Results:**

We finally included 300 patients (mean age: 51 to 66.6 years; male: 182; female: 118) from 9 studies of hepatic malignant tumors. The meta-analysis showed that comparing with the initial values, the longest diameter of the tumors was significantly decreased at the last follow-up months after IRE. Furthermore, the ALP, AST and total bilirubin levels were increased at 1 day after IRE while returned to baseline at the last follow-up month. No risk of publication bias was found, and all literatures were assessed good quality according to NOS.

**Conclusions:**

The pooled data indicated that IRE could be a minimal invasive and effective approach for patients who had preoperative poor liver function or those whose masses were in refractory locations where surgical resection was unsuitable.

## INTRODUCTION

Cancer became a main threat to public health worldwide, and incidence rates have risen in most countries since 1990. Liver cancer ranked 6th and 3rd of the list for cancer incidence and cancer death in 2013, respectively. It was ranked 11th and 7th respectively for incidence and mortality in developed countries while 5th and 2nd in developing countries. In 2013, the disease resulted in 20.9 million disability adjusted life years (DALYs), with 14% in developed countries and 86% in developing countries. Liver cancer was usually diagnosed cancer and main cause of cancer death in 2013 for men in the areas such as Africa, South Asia and Mongolia. Similarly, this also happened for women in 2013 in Mongolia. It was reported approximately 792000 new cases of liver cancers and 818 000 deaths in 2013 in the world [1]. Long-term alcohol consumption, hepatitis B virus (HBV) and C (HCV) are all the risk factors linked with hepatocarcinogenesis [2, 3]. Therapy of hepatocellular carcinoma (HCC) could be curative, palliative, and symptomatic, which included surgery, transplantation, and local tumour ablation [4, 5].

Traditionally, thermal ablation techniques like radiofrequency ablation (RFA) and microwave ablation (MWA) kill target lesions by gathering thermal energy. Major limitations included low power, shaft heating, large diameter probes, and not being able to effectively treat tumors adjacent to a major blood vessel due to the “heat sink” effect and relapse easily [6–8]. It was reported a high recurrence rate from 4.6% to 48% after the thermal ablations for patients with hepatic cancer or hepatic metastases [9–11]. Unlike these existing thermal ablation techniques, irreversible electroporation (IRE) is a new nonthermal ablative technique, which triggers cell death by altering the permeability of the cellular membrane based on the pulsed direct current, and destroying the lipid bilayer integrity to enable molecules through the cell membrane [12–15]. IRE can induce tissue necrosis within micro- to millisecond ranges while conventional ablation techniques need almost from 30 minutes to hours. It also generated a clear boundary between the ablated and unablated area *in vivo* [16]. In the basis of these reasons, IRE could be an effective treatment option for target tumors in challenging locations of porta hepatis, gallbladder, bile ducts, pancreatic duct and ureter [17–22].

However, in recent years, numerous emerging reports, which focused on the change of the longest diameter and laboratory indexes of patients with liver cancer pre- and post-IRE [13, 15, 23–29], are still inconclusive how the variation of these parameters are. Therefore, we performed a systematic review and meta-analysis to enable a more precise evaluation of the outcomes that correlated a response to IRE.

## RESULTS

### Study characteristics

**Figure 1:**
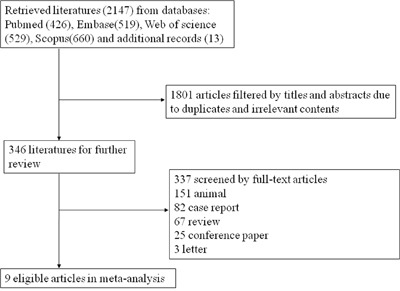
Flow diagram of the study selection process

We finally identified from 9 studies of hepatic malignant tumors representing a total of 300 patients (male: 182; female: 118). Two thousand one hundred and thirty-eight studies were ineligible for inclusion according to predefined search strategies until July 26th, 2016 (Figure 1). Table 1 summarized the characteristics of the 7 retrospective and 3 prospective cohort studies with the mean follow-up between 2 and 23 months. The mean age of included participants ranged from 51 to 66.6 years. The treated tumors in these studies of recent decades mainly included primary and metastatic hepatic disease, in which US/CT-guided percutaneous IRE became the main treatment methods. Furthermore, it showed IRE had a few major complications including 4 hepatic abscesses, 1 bile duct dilatation, 1 arrhythmia, 1 portal vein thrombosis in Table 1 and these patients in this study had a better prognosis. Most of these observational studies were of good quality ranging from 6 to 9 scores using the Newcastle-Ottawa scale. Risk of bias was described in Supplementary Table 1.

**Table 1:** Summary of the included studies

Author	Year	Study period	Design style	Country	Population characteristics	Treatment methods	Patients (No. of benign thyroid nodules)	Male/female	Age(years)	Follow-up interval(months)	Complication	Imaging methods	Prognosis	NOS score
Dollinger M et al.	2016	NA	Retrospective cohort	Germany	7 HCC;2 cholangiocellular carcinoma;10 metastatic colorectal tumor,2 metastatic breast carcinoma,3 others	CT-guided percutaneous IRE	26(53)	17/9	59.3±11.2(37-77)	7.2±5.3	NA	MR	Local tumor recurrence:18.2%	8
Padia SA et al.	2016	2011.4-2013.12	Retrospective cohort	USA	20 HCC	US/CT-guided percutaneous IRE	20(20)	14/6	62(50-76)	12	NA	MR	Primary efficacy rate:90%	7
Barabasch A et al.	2016	2012-2015	Prospective cohort	Germany	15 colorectal cancer;4 breast cancer;2 pancreatic cancer;2 esophageal carcinoma;1 melanoma;1 mesothelioma;1 RCC;1 GIST	CT-guided percutaneous IRE	27(37)	13/14	62±11(46-68)	23±11	NA	MR	CR:57%	7
Sugimoto K et al.	2015	2014.1-2014.6	Prospective cohort	Japan	5 HCC	US-guided percutaneous IRE	5(6)	3/2	66.6±5.8	8	No	CT/MR/US	2 new lesions	6
Niessen C et al.	2015	2011.12-2013.3	Prospective cohort	Germany	22 HCC;6 cholangiocellular carcinoma;16 colorectal metastasis;4 other metastasis	US/CT-guided percutaneous IRE	25(48)	21/4	59.4±11.2	6	NA	MR	Local recurrence rate:29.2%	7
Froud T et al.	2015	2010.1-2014.10	Retrospective cohort	USA	62 metastatic disease;53 hepatocellular carcinoma;8 cholangiocarcinoma;1 unknown	CT-guided IRE	124	71/53	59.8±11.4	2	NA	PET	NA	9
Dollinger M et al.	2014	NA	Retrospective cohort	Germany	14 HCC;11 colorectal tumor;5 cholangiocellular carcinoma;1 seminomatous testicular tumor;1 esophageal carcinoma;1 neuroendocrine tumor;1 other	CT-guided percutaneous IRE	34(52)	28/6	64(22-80)	4.7(0.3-17)	4 hepatic abscesses	CT	Reduction in volume to 29% of initial value	7
Silk MT et al.	2014	2011.1-2012.9	Retrospective cohort	USA	16 metastatic colorectal carcinoma;5 metastatic pancreatic carcinoma;1 metastatic hemangiopericytoma	CT or PET/CT percutaneous IRE	11(22)	4/7	60(45-81)	9±6	1 bile duct dilatation	CT	Local tumor recurrence:54.5%	6
Kingham TP et al.	2012	2011.1.1-2011.11.2	Retrospective cohort	USA	21 metastatic colorectal cancer;2 HCC;2 metastatic pancreatic neuroendocrine;1 metastatic ampullary carcinoma;1 hemangiopericytoma;1 leiomyosarcoma metastasis tumor	US/CT-guided open/percutaneous IRE	28(65)	11/17	51(32-81)	6(1-9)	1 intraoperative arrhythmia;1 postoperative portal vein thrombosis	CT/MR	Persistent disease rate:1.9%;local recurrence rate:5.7%	7

NA:not available;

CR:complete response;

RCC: renal cell carcinoma;

GIST: gastrointestinal stromal tumor;

HCC: hepatocellular carcinoma;

US:ultrasound;

CT: Computed Tomography;

MR: Magnetic Resonance;

NOS: Newcastle-Ottawa Scale.

### Meta-analysis results

### Heterogeneity test result

Results of this meta-analysis showed that comparing with the initial values, the longest diameter of the tumors was significantly decreased at the last follow-up month after IRE (1 month, SMD 95%CI: 0.447(0.189-0.704)). Furthermore, the ALP, AST and total bilirubin levels were increased at 1 day after IRE while unchanged at the last follow-up month (Table 2, Supplementary Figure 1-4) (ALP: 1 day, SMD 95%CI: -0.5(-0.88--0.12); the last follow-up month, SMD 95%CI: 0.228(-0.223-0.679); AST: 1 day, SMD 95%CI: -2.82(-4.296-1.343); the last follow-up month, SMD 95%CI: -0.028(-0.511-0.454); total bilirubin: 1 day, SMD 95%CI: -0.902(-1.254--0.551); the last follow-up month, SMD 95%CI: -0.131(-0.551-0.288)).

**Table 2:** Subgroup analysis of the outcomes of IRE for hepatic malignant tumors

Subgroup	Number of studies	SMD (95%CI)	Z score	*p*	Heterogeneity test			Publication bias		
					Q	τ^2^	*I^2^*(%)	*p*	*t*	*p*
Largest diameter	5	0.447(0.189-0.704)	3.4	0.001	7.86	NA	49.1	0.097	-0.9	0.434
ALP										
1 day	3	-0.5(-0.88--0.12)	2.58	0.01	4.17	NA	52.1	0.124	-0.51	0.7
Last month	2	0.228(-0.223-0.679)	0.99	0.322	3.79	NA	73.6	0.051	NA	NA
AST										
1 day	3	-2.82(-4.296-1.343)	3.74	<0.001	17.41	1.3403	88.5	<0.001	-1.9	0.309
Last month	2	-0.028(-0.511-0.454)	0.12	0.908	0.08	NA	0	0.784	NA	NA
Total bilirubin										
1 day	4	-0.902(-1.254--0.551)	5.03	<0.001	0.1	NA	0	0.991	-1.56	0.259
Last month	3	-0.131(-0.551-0.288)	0.61	0.54	0.76	NA	0	0.685	-0.94	0.521

ALP: alkaline phosphatase; AST: aspartate aminotransferase; SMD: standardized mean difference; CI: confidence interval.

SMD test was estimated by Z score. In SMD test, *p*<0.05 indicates the result significant.

Heterogeneity was appraised by Q, *τ^2^* and *I^2^* statistics. Q value indicates random error; Tau2 (*τ^2^*) value means the variations between studies; *I^2^* value represents the percentage of inter-study difference in the overall heterogeneity.

### Sensitivity analyses and publication bias

Assessment of the subgroup analysis were generally in accordance with the sensitivity analysis. No evidence of publication bias was found by means of funnel plot asymmetry and Egger's linear regression test (Table 2).

## DISCUSSION

Liver cancer contributed a lot to the total burden of cancer, which ranked the third in most common causes of cancer death worldwide. The incidence was still highest in the developing world while rising in the developed world [30]. The pooled data of meta-analysis showed the increased ALP, AST and total bilirubin levels at 1 day after IRE while decreased longest diameter, unchanged ALP, AST and total bilirubin levels at the last follow-up months. Although there were a small number of complications such as arrhythmia, portal vein thrombosis, bile duct dilatation and hepatic abscesses, it suggested that IRE may be a potential candidate of primary or metastatic liver malignancies.

Our study showed that the longest diameter was decreased a few months later. In previous study, it showed after about 4.7 months follow-up, CT scans found a decrease in volume to 29% and in diameter to 61% comparing with the initial values [27]. A retrospective study by MR imaging appearances of treated tumors revealed there was a decrease of 28.9% in size of the ablation zone at day 90 versus day 1 after IRE [13], which was similar to another systematic longitudinal study [24]. However, a research of 24 HCC lesions in 20 patients showed no significant difference in the largest diameter during the 18-month period [31]. Nevertheless, the curative effect of IRE was still attractive. Recent studies have detected the increased plasma levels of IL-6 and IL-10 induced by different ablation modalities [32]. Continuous opening of microvessels in the coagulated zone from IRE enlarged the area and accumulation of infiltrative cells, leading to more robust systemic reactions including tumorigenic and immunogenic effects compared to RFA [33]. Furthermore, it was reported that cell death of IRE has enhanced BAX (BCL-2) staining comparing with non-ablated area, which suggested the impact of electroporation on the apoptosis rather than RFA on the thermal coagulative necrosis [34]. The cellular tissue could be repaired, which was observed in the pathologic analysis showing hepatocyte proliferation about 24 hours after ablation. It may be that apoptotic cells were quickly eliminated by phagocytosis and replaced with innate cellular regeneration [35]. These may lead to the decreased masses and indicated that IRE is a promising approach to deal with hepatic malignant tumors.

Besides the longest diameter in our study, rapid elevations after IRE were seen within 24 hours in the liver transaminases of ALP and AST, which were signals of hepatocellular injury or necrosis. It was reported that AST locating in hepatocytes leaked out into the bloodstream due to the hepatocellular damage [36]. In this study, the transaminases returned to baseline following one or two months. Serum bilirubin also rose to peak level on day 1 and normalized two months later. The peak values of elevation were consistent with those of other ablation modalities. Previous study showed that the change in AST levels after cryotherapy of liver tumors was associated with the thrombocytopenia level and could be a potential early signal of severe thrombocytopenia [37] while ALP levels were the most sensitive biochemical marker of cholestasis [38]. Thus physicians could not be frightened by the early rapid elevation in AST, ALT and total bilirubin levels in the event of hepatocellular damage and cell death after IRE, of which postoperative regular follow-up should be necessary.

Several limitations were arisen from this study, perhaps the most important of which was the limited sample that has different hepatic tumor types with various histological characteristics might have impacts on the treatment response. Secondly, we conducted this study based on the different imaging methods such as CT, MRI and ultrasonography, which may cause the measuring error. Thirdly, some drugs of anesthetic agents like halothane; antibiotics like amoxicillin, ciprofloxacin; and analgesics like acetaminophen could have toxic effects on the liver by directly influencing hepatocytes or regulating an immune response [39].

In spite of these limitations, the meta-analysis provided evidence that IRE might be effective for patients with challenging hepatic malignant tumors. All published literatures about this subject were seriously searched and cross-checked by two individual investigators through relevant protocols. We also carefully extracted and combined data in each study to provide a robust evaluation of post-IRE outcomes.

## MATERIALS AND METHODS

### Search strategy

This meta-analysis was seriously performed according to the PRISMA statement [40]. We searched all studies based on PubMed, Embase, Web of science and Scopus from database inception to July 26th, 2016, where the keywords “irreversible electroporation”, “IRE”, “nanoknife”, “liver”, “hepartic” were used. Detailed search strategies for each database were available in Supplementary File. We also earnestly checked references in the included studies of other potential articles.

### Inclusion criteria

We selected studies according to the criterias as follows: 1) original article; 2) prospective or retrospective studies, including cohorts and trials; 3) primary or metastatic liver malignancies treated with percutaneous IRE; 4) it provided sufficient data to calculate these estimates including the longest diameter, alkaline phosphatase (ALP), aspartate aminotransferase (AST) and serum total bilirubin.

### Exclusion criteria

These studies would be ineligible with the criterias below: 1) case reports, case series, and animal studies; 2) benign hepatic lesions; 3) If studies had multiple reports, the latest or most complete article was retained; 4) patients with pacemakers, a history of cardiac arrhythmias, metastases in several other organs, and extremely large lesions [6].

### Data extraction and literature quality assessment

All the retrieve data were imported into reference management software (Endnote X7, free trial version, Zhejiang University School of Medicine, Hangzhou, China) after the electronical or manual removal of duplicate citations. The rest articles were screened and checked by two individual investigators according to the pre-defined criteria. The information extracted from each paper included author, publication year, study period, design style, country, population characteristics, treatment methods, number of the tumor, male or female number, age, follow-up interval, complication, imaging methods, prognosis and NOS score. We also attempted to email to authors for additional information if necessary. Disagreements were discussed to be determined with a third reviewer. In addition, two investigators independently cross-checked the risk of bias using the Newcastle-Ottawa Scale for observational studies.

### Statistical analysis

Comparing with the initial values, the meta-analysis checked the changes of the longest diameter, alkaline phosphatase (ALP), aspartate aminotransferase (AST) and serum total bilirubin at 1 day and the last follow-up months after IRE. For each study, we estimated standard mean difference (SMD) and its 95% CIs for each outcome. We estimated the heterogeneity across studies using the Q statistic [41], *τ^2^* and *I^2^* = 100%×(Q-df)/Q [42]. If a two-sided *p* value was less than 0.05 considered as statistically significant, then a random-effect model was used. Otherwise, a fixed-effect model would be applied. In addition, sensitivity analysis was used to appraise the impact of the remaining studies without the larger one's effect. We calculated the effect of publication bias by the Egger regression asymmetry test and funnel plots [43]. In this study, Stata 12.0 software (Stata Corp, College Station, Texas) was conducted for all statistical analyses.

## CONCLUSION

In conclusion, our data suggested that IRE, based on its advantage of minimal invasive approach, could be a candidate for patients who had preoperative poor liver reserve capacity or those whose masses were in refractory locations where surgical resection was unsuitable and devastative. And furthermore, more prospective large-scale studies with the long-term follow-up should be performed to confirm this in the future.

## SUPPLEMENTARY MATERIALS FIGURES AND TABLES



## Abbreviations

IRE: Irreversible electroporation
SMD: standard mean difference
CI: confidence interval
ALP: alkaline phosphatase
AST: aspartate aminotransferase
NOS: Newcastle-Ottawa Scale
DALYs: disability adjusted life years
HBV: hepatitis B virus
HCV: hepatitis C virus
HCC: hepatocellular carcinoma
RFA: radiofrequency ablation
MWA: microwave ablation
PRISMA: Preferred Reporting Items for Systematic Reviews and Meta-Analyses
IL-6: interleukin-6
BCL-2: B-cell lymphoma-2
