# Supplementary material for: Ablation of hepatic malignant tumors with irreversible electroporation: A systematic review and meta-analysis of outcomes
Source: Oncotarget. 2016 Dec 20;8(4):5853–60. doi: 10.18632/oncotarget.14030 (PMC5351595; doi:10.18632/oncotarget.14030)
Supplement: Supplementary file 1 [file oncotarget-08-5853-s001.pdf]

Ablation of hepatic malignant tumors with irreversible electroporation: A systematic review and meta-analysis of outcomes

SUPPLEMENTARY DATA

Search strategy

Pubmed (1950-present)

1. 'irreversible electroporation' OR IRE OR nanoknife
2. liver OR hepar OR hepatic
3. 1 AND 2
4. 'electroporation'[Mesh]
5. 'liver' [Mesh]
6. 4 AND 5
7. 3 OR 6

Embase(1980-present)

1. 'irreversible electroporation':ab,ti
2. 'IRE':ab,ti
3. 'nanoknife':ab,ti
4. 1 OR 2 OR 3
5. 'liver':ab,ti
6. 'hepar':ab,ti
7. 'hepatic':ab,ti
8. 5 OR 6 OR 7
9. 4 AND 8

Scoups

1. TITLE-ABS-KEY ('irreversible electroporation')
2. TITLE-ABS-KEY ('IRE')
3. TITLE-ABS-KEY ('nanoknife')
4. 1 OR 2 OR 3
5. TITLE-ABS-KEY ('liver')
6. TITLE-ABS-KEY ('hepar')
7. TITLE-ABS-KEY ('hepatic')
8. 5 OR 6 OR 7
9. 4 AND 8

Web of science

1. TS=(electroporation)
2. TS=(liver)
3. 1 AND 2
4. TI=(irreversible electroporation)
5. TI=(IRE)
6. TI=(nanoknife)
7. 4 OR 5 OR 6
8. TI=(liver)
9. TI=(hepar)
10. TI=(hepatic)
11. 8 OR 9 OR 10
12. 7 AND 11
13. 3 OR 12

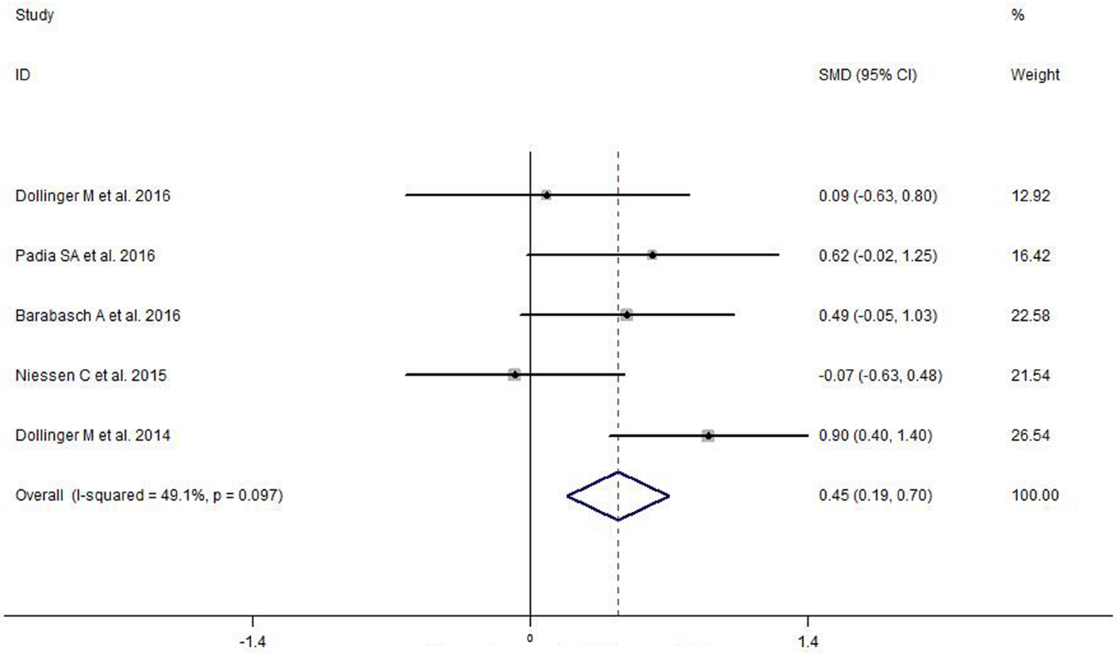

Supplementary Figure 1: Longest diameter change of tumors before and last follow-up month after IRE treatment. Comparing with the initial largest diameter, the post-IRE longest diameter showed the decreased response at last follow-up month after IRE treatment.

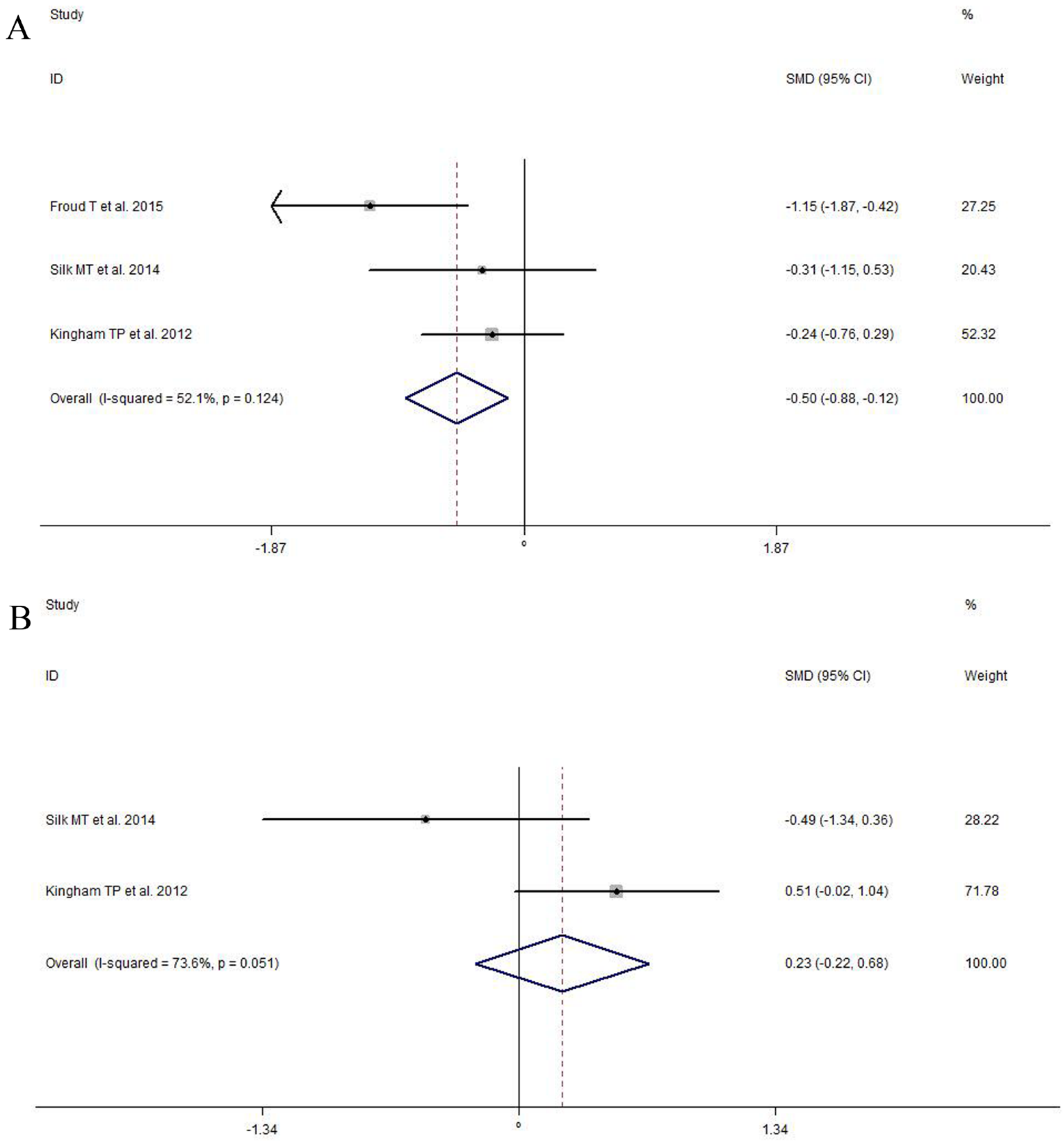

Supplementary Figure 2: ALP level change before and 1 **A.** and last follow-up month **B.** after IRE treatment. Comparing with the initial ALP level, the post-IRE ALP level increased at 1 day after IRE while unchanged at the last follow-up month.

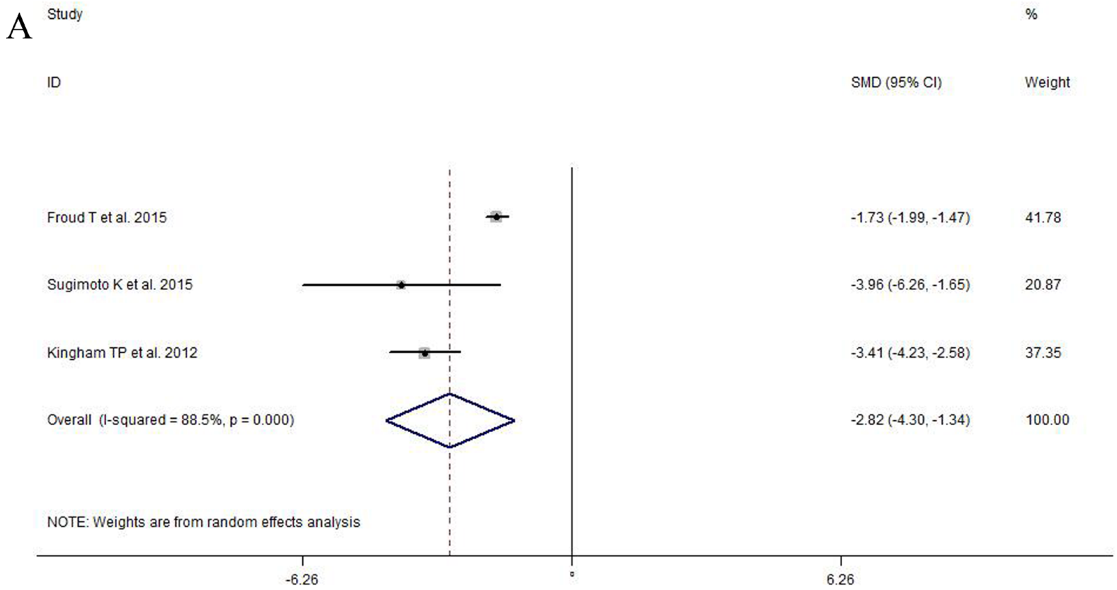

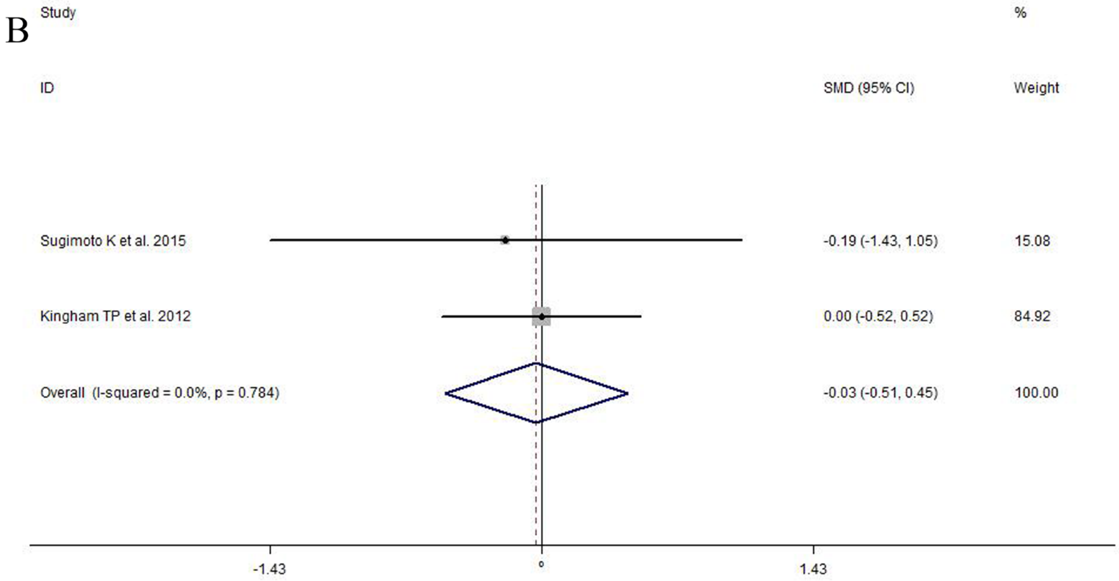

Supplementary Figure 3: AST level change before and 1 **A.** and last follow-up month **B.** after IRE treatment. Comparing with the initial AST level, the post-IRE AST level increased at 1 day after IRE while unchanged at the last follow-up month.

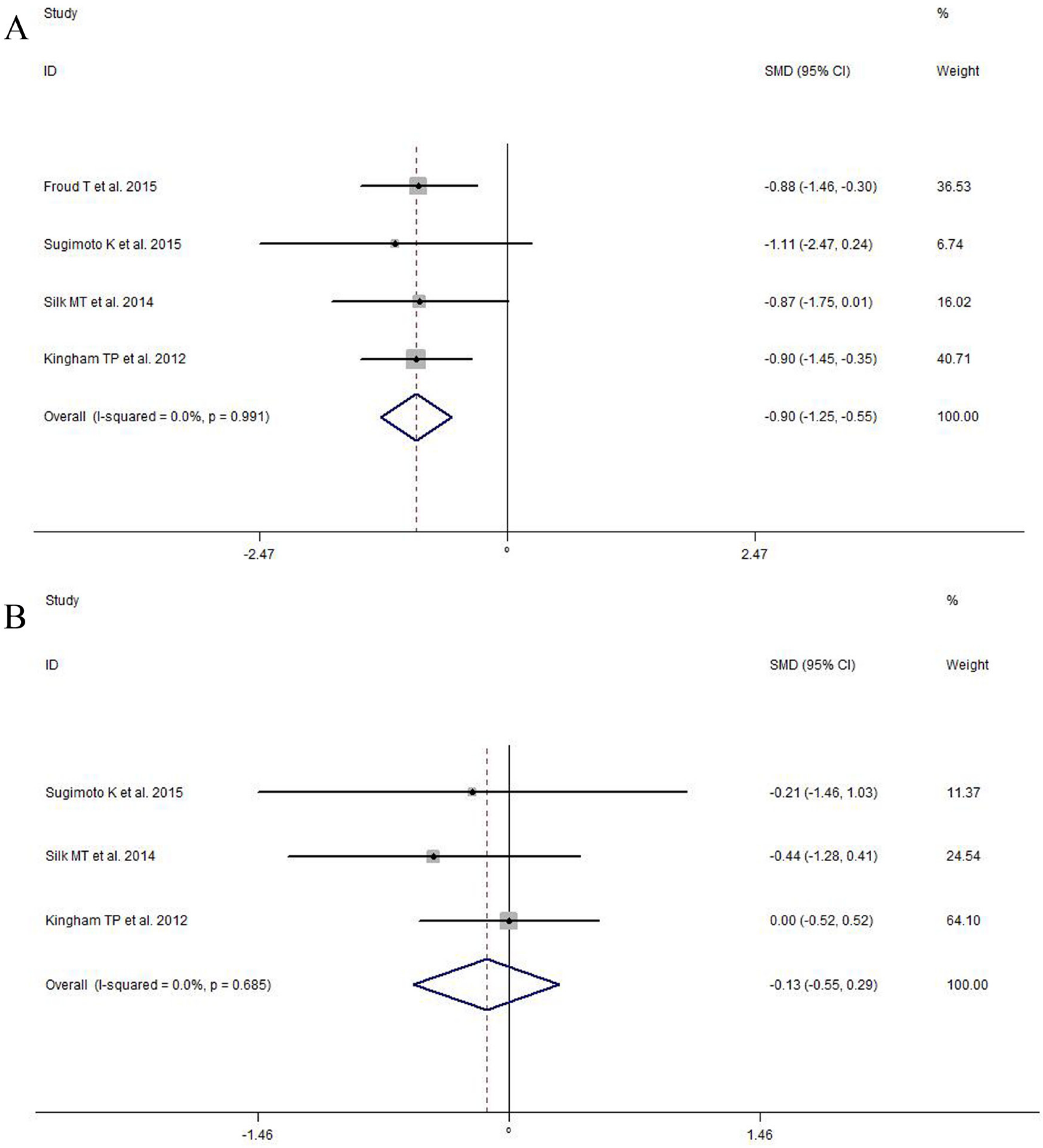

Supplementary Figure 4: Serum total bilirubin level change before and 1 **A.** and last follow-up month **B.** after IRE treatment. Comparing with the initial total bilirubin level, the post-IRE total bilirubin level increased at 1 day after IRE while unchanged at the last follow-up month.

Supplementary Table 1: NOS for the quality of the included literatures

Study	Selection				Comparability	Exposure			
	Sufficient definition of cases	Representativeness of cases	Selection of controls	Definition of controls	Comparability of cases and controls based on the design or analysis	Ascertainment of exposure	Same method of ascertainment for cases and controls	Nonresponse rate	Total scores
Dollinger M et al. 2016	*	*	*	*	*	*	*	*	8
Padia SA et al. 2016	*	*	*	*	*	*	*	NA	7
Barabasch A et al. 2016	*	*	*	*	*	*	*	NA	7
Froud T et al. 2015	*	*	*	*	**	*	*	*	9
Sugimoto K et al. 2015	*	*	*	*	0	*	*	NA	6
Niessen C et al. 2015	*	*	*	*	*	*	*	NA	7
Silk MT et al. 2014	*	*	*	*	0	*	*	NA	6
Dollinger M et al. 2014	*	*	*	*	*	*	*	NA	7
Kingham TP et al. 2012	*	*	*	*	*	*	*	NA	7

NOS: Newcastle-Ottawa Scale;
NA:not available.
